# To what do psychiatric diagnoses refer? A two-dimensional semantic analysis of diagnostic terms

**DOI:** 10.1016/j.shpsc.2015.10.001

**Published:** 2016-02

**Authors:** Hane Htut Maung

**Affiliations:** Department of Politics, Philosophy, and Religion, Lancaster University, Lancaster LA1 4YL, United Kingdom

**Keywords:** Diagnosis, Medicine, Psychiatry, Causal theory of reference, Two-dimensional semantics

## Abstract

In somatic medicine, diagnostic terms often refer to the disease processes that are the causes of patients' symptoms. The language used in some clinical textbooks and health information resources suggests that this is also sometimes assumed to be the case with diagnoses in psychiatry. However, this seems to be in tension with the ways in which psychiatric diagnoses are defined in diagnostic manuals, according to which they refer solely to clusters of symptoms. This paper explores how theories of reference in the philosophy of language can help to resolve this tension. After the evaluation of descriptive and causal theories of reference, I put forward a conceptual framework based on two-dimensional semantics that allows the causal analysis of diagnostic terms in psychiatry, while taking seriously their descriptive definitions in diagnostic manuals. While the framework is presented as a solution to a problem regarding the semantics of psychiatric diagnoses, it can also accommodate the analysis of diagnostic terms in other medical disciplines.

## Highlights

• Descriptive and causal theories of reference are evaluated regarding diagnoses.
• A two-dimensional semantic framework analysis of diagnostic terms is proposed.
• This resolves tension between the uses and definitions of psychiatric diagnoses.

When citing this paper, please use the full journal title *Studies in History and Philosophy of Biological and Biomedical Sciences*

## 1 Introduction

Diagnoses are central to the practice of medicine. In addition to predictive, therapeutic, and social functions, diagnoses often serve as causal explanations of patients' symptoms. The diagnosis of appendicitis, for example, explains why the patient has abdominal pain by conveying information about what is causing the abdominal pain. The ways in which psychiatric diagnoses are sometimes described in clinical texts suggest that some clinicians also consider them to refer to the causes of symptoms. For example, the *Oxford Handbook of Clinical Specialties* states: “Most auditory hallucinations not associated with falling asleep or waking up are caused by schizophrenia or depression” (Collier, Longmore, & Amarakone, 2013, p.317). Similarly, the following sentence is from NHS Choices, one of the leading health information websites for the general public in the United Kingdom: “Depression affects people in different ways and can cause a wide variety of symptoms” (NHS Choices, 2014, Introduction, para. 5). These passages show that psychiatric diagnoses are often communicated to clinicians and the public as if they refer to the causes of their symptoms, much like appendicitis refers to a cause of abdominal pain.

However, this contrasts with how psychiatric diagnoses are defined. According to the most recent editions of the American Psychiatric Association's (APA) *Diagnostic and Statistical Manual of Mental Disorders* (DSM), the dominant classification system in psychiatry, psychiatric diagnoses are defined through their symptoms. For example, the current edition, DSM-5, defines delusional disorder as follows: “The essential feature of delusional disorder is the presence of one or more delusions that persist for at least 1 month” (APA, 2013, p.92). Similarly, the definition of major depressive disorder includes the following: “The essential feature of a major depressive episode is a period of at least 2 weeks during which there is either depressed mood or the loss of interest or pleasure in nearly all activities” (APA, 2013, p.163).

Hence, there are two kinds of talk going on regarding psychiatric diagnoses: diagnoses are used to refer to the causes of symptoms, yet they are defined descriptively through these symptoms. From a social perspective, this ambiguity is not entirely surprising. As argued by Haslanger (2006), a term can express different concepts to fulfil different ideological functions in different contexts. For instance, she observes that “parent” is often defined as “immediate progenitor”, but used in some contexts to mean “primary caregiver”. She respectively terms these the manifest concept and the operative concept, and suggests that the divergences between the two can help reveal the ideological function of a term, as well as open up the manifest concept to normative critique. The divergences between the definitions and uses of psychiatric diagnoses, then, might reflect the social expectations for a diagnosis to function both as a label for certain kinds of behaviour and as a scientific explanation of certain distressing symptoms.

However, from an epistemological standpoint, I argue that the ambiguity regarding diagnostic terms is problematic. First, in the case of psychiatric diagnoses, the two kinds of talk are in tension. At least since Hume's analysis of causation, it has generally been accepted in philosophy that causes are distinct from their effects. While it is possible, indeed common, for someone to be both the immediate progenitor and the primary caregiver of a child, a set of symptoms cannot be its own cause. Therefore, if psychiatric diagnoses refer to clusters of symptoms as suggested by the DSM-5 definitions, then they cannot refer to the causes of these symptoms. Second, although it is certainly the case that terms can express different things in different contexts, the two kinds of talk regarding psychiatric diagnoses often occur within the same context. “Parent” can be taken to mean “immediate progenitor” or “primary caregiver”, depending on whether one is defining the term in a biological context or whether one is a teacher writing parents' evening invitations, but the same psychiatrist who uses a diagnosis to pick out a set of symptoms may also invoke it as a causal explanation of the symptoms within the same clinical encounter.

This does not only present an epistemological problem, but one that has inspired criticism of psychiatric practice. Szasz, a major proponent of the 1960s antipsychiatry movement, criticised the way in which mental illness is reified as a cause of certain types of behaviour when it is only supposed to be a shorthand label for these types of behaviour (Szasz, 1961, p.15). There are also implications at the level of everyday doctor–patient communication. Given the historical and cultural underpinnings of psychiatry as a scientific discipline, there is an expectation for its diagnostic terms to pick out actual disease processes in the world that help to explain symptoms, as evidenced by the language used in clinical texts. However, if psychiatric diagnoses merely refer to sets of symptoms as suggested by the DSM-5 definitions, then offering these diagnoses as explanations of the symptoms amounts to a tautology. This raises the ethical worry that patients are misled into believing that their symptoms are being explained by their diagnoses, when they are merely being labelled.

This paper explores how theories of reference in the philosophy of language can help to clarify the semantics of diagnostic terms in psychiatry. My aim is to present a conceptual framework that resolves the epistemological tension between their uses and definitions. This is not intended to be a normative account of what psychiatric practice should be like, but rather a description of the semantic practices of psychiatrists that accommodates the actuality of diagnostic terms being used in two seemingly contradictory ways. I begin by considering the traditional view that some diagnostic terms describe symptoms while others refer to causes, and that the development of a diagnostic term involves a progressive change in its conception from the former to the latter. I discuss the worry that this change implies incommensurability between older and newer conceptions of a diagnostic term. I then look at the causal theory of reference as a more reasonable account of diagnostic terms that avoids the implication of semantic incommensurability. Despite its advantages, I argue that something more than a pure causal theory of reference is required for an adequate analysis of psychiatric diagnoses. I put forward a solution based on the conceptual framework of two-dimensional semantics, which allows diagnostic terms to refer to the causes of symptoms despite being defined through their symptoms.

## 2 Descriptive and causal conceptions of diagnostic terms

### 2.1 Ontological descriptivism

In her paper, “Is This Dame Melancholy?” (2003), Radden contrasts descriptive and causal approaches to defining disorders. The descriptive approach provides definitions of disorders that consist of descriptions of symptoms, without mention of the processes that cause these symptoms. Radden observes that this is the approach used by the most recent editions of DSM to define psychiatric disorders. Descriptive definitions also sometimes feature in other medical disciplines. The definition of “chronic bronchitis”, for example, includes “cough and sputum expectoration on most days for at least three months of the year and for at least two consecutive years” (Braman, 2006, p.104).

Radden relates the descriptive approach to a view she calls ontological descriptivism, which suggests that diagnostic terms refer exclusively to clusters of symptoms. According to this view, “major depressive disorder” refers solely to the conjunction of the patient's low mood, loss of interest, and other associated symptoms. This is not to say that this conjunction of symptoms does not have a cause, but merely that the diagnosis of major depressive disorder does not refer to any causes. According to this analysis, clinical texts are wrong when they cite psychiatric diagnoses as referring to the causes of certain symptoms. Rather, they refer to the symptoms themselves.

In contrast to descriptivism, a causal conception of diagnostic terms states that a diagnosis does not refer to the conjunction of symptoms, but to its cause. For example, “appendicitis” does not refer to abdominal pain and associated symptoms. Rather, it refers to the cause of these symptoms, namely inflammation of the appendix.

### 2.2 Conceptual change

It has been suggested that the historical development of a diagnostic term involves a progressive change from descriptive to causal conceptions. Hempel (1965) and Thagard (1999) propose that a scientific discipline proceeds from an early observational stage, when the aim is to describe the phenomena being studied, to later theoretical stages, when the aim is to explain the phenomena with appeal to theories.^1^ Accordingly, Hempel predicted that the classification of psychiatric disorders will follow this trend from descriptive to progressively more theoretical language.

It is also worth noting that diagnostic terms can undergo other sorts of conceptual change in addition to the sort proposed by Hempel and Thagard. For example, there may be a change in the descriptive definition of the disorder, such as when the symptom criteria for schizophrenia were modified between the publications of DSM-IV (APA, 1994) to DSM-5 (APA, 2013). There may be a change from one causal conception to another, such as when Creutzfeldt-Jakob disease went from being considered a disease caused by slow viruses to a disease caused by prions.

The image of disease understanding proposed so far suggests that diagnostic terms undergo conceptual changes throughout their histories. According to Hempel and Thagard, a diagnostic term normally begins as a descriptive concept that refers to a set of associated symptoms. As the aetiology and mechanisms underlying these symptoms are discovered, it becomes a concept that refers to what normally causes these symptoms. This suggests that psychiatric diagnoses do not currently refer to the causes of their symptoms, but that there is hope that they will in the future, as our disease understanding increases.

### 2.3 Semantic incommensurability

The move from descriptive to causal conceptions of diseases is generally considered to be positive, as it allows greater explanatory power, more accurate prediction, improved prevention, and the development of targeted treatments. However, this conceptual change further complicates the question of what states of affair diagnostic terms refer to. It seems to suggest that a diagnostic term can refer to a conjunction of symptoms at one time and refer to what causes these symptoms at a later time.

There is disagreement among philosophers over whether this is rational conceptual change or whether it amounts to a more serious problem of semantic incommensurability. Thagard (1999) acknowledges that conceptual change does occur with changes in disease understanding, but presents this as being largely unproblematic. However, there are philosophers who propose that this kind of conceptual change implies radical incommensurability between the old and new concepts. Notably, Kuhn, 1962, Kuhn, 2000 and Feyerabend (1962) insist that the gap of meaning between old and new conceptions of a term amounts to linguistic instability, thus precluding meaningful comparison between the term's uses before and after the conceptual change.

The worry about semantic incommensurability regarding diagnostic terms goes at least as far back as Fleck's *Genesis and Development of a Scientific Fact* (1981 [1935]). Using the example of syphilis, Fleck argues that new concepts of a disease are not adequate substitutes for the old concepts. Throughout its history, “syphilis” had been defined as a disease that is treated by mercury, a set of characteristic symptoms, and then finally as *Treponema pallidum* infection. These different concepts have different extensions, and so cannot be equated. For example, “a disease that is treated by mercury” excludes treatment-resistant cases of *T. pallidum* infection and “a set of characteristic symptoms” excludes asymptomatic cases.

Radden (2003) reaches a similar formulation in her cross-historical comparison of pre-nineteenth century melancholia and today's depression. Here, the comparison is between two descriptive conceptions of what is often assumed to be the same disorder: “melancholia” and “depression” are often thought to refer to the same thing. However, Radden argues that they cannot be equated. First, there are differences between the symptom profiles of melancholia and depression, which suggests that the two are not coextensive. For instance, Radden observes that some cases of modern-day schizophrenia and obsessive-compulsive disorder would also qualify as cases of melancholia. Second, if the descriptivism of the DSM is assumed and “depression” refers exclusively to a set of symptoms, then depression and melancholia cannot be equated on causal grounds, because causal factors are not part of the meaning of “depression”.

Semantic incommensurability challenges the intuition that there is continuity between the past and present concepts of a disease. As previously noted, “syphilis” had been defined as a set of characteristic symptoms before it was later defined as *T. pallidum* infection. If, after the discovery of *T. pallidum*, it turns out that some of the previous cases diagnosed as syphilis on the basis of their symptoms were not caused by *T. pallidum*, then there is an intuition that such cases were false positives and that it turned out that they were not actually cases of syphilis. We might say that the doctors who identified such cases as syphilis turned out to be wrong. However, the incommensurability problem suggests that we could not claim that they were wrong, because they were using a different meaning of “syphilis”. With respect to their meaning of “syphilis”, they were right.

This is untenable, because it seems to suggest that discoveries in medical science do not actually increase our understanding of individual diseases. LaPorte (2004, p.114) notes that what appears to be an increase in understanding of a term is actually a case of changing its meaning, so that it refers to a different state of affairs. Before the discovery of *T. pallidum*, “syphilis” used to refer to a set of characteristic symptoms. After the discovery of *T. pallidum*, it referred to *T. pallidum* infection. Let us call these concepts SYPHILIS-1 and SYPHILIS-2, respectively. Rather than resulting in an increase in the understanding of SYPHILIS-1, the discovery of *T. pallidum* resulted in “syphilis” being displaced from SYPHILIS-1 and attached instead onto SYPHILIS-2. Similarly, Sankey (2009, p.198) notes that if a later concept does not refer to the same phenomenon to which an earlier concept had referred, then the conceptual change does not constitute an increase in knowledge about the phenomenon referred to by the earlier concept.

The implication of incommensurability not only makes cross-historical comparisons of disorders problematic, but also cross-cultural comparisons. As noted by Radden (2003, p.44), it is often reported that people with depression in China present with different symptoms from people with depression in the West. In particular, Chinese depression is said to present predominantly with somatic symptoms such as back pain and headache, rather than mood symptoms. Radden even notes that in some cases, there is no apparent commonality between the symptoms of Chinese and Western depression. Again, she argues that if descriptivism is assumed and “depression” is defined exclusively through its symptoms, then Chinese and Western depression cannot be equated because of this lack of commonality between their symptom profiles.

## 3 The causal theory of reference

### 3.1 A solution to incommensurability

In contemporary discussions (LaPorte, 2004, Sankey, 2009), semantic incommensurability is normally presented as being a problem for analyses of conceptual change that presume the descriptive theory of reference. The descriptive theory, as advocated by Frege (1952 [1892]) and Russell (1905), states that the sense, or intension, of a term consists of a description. The reference, or extension, of the term is what satisfies this description. However, as disease understanding changes, so does the description associated with a diagnostic term. Under the descriptive theory, this change in description amounts to a change in reference, as is suggested by the above case of syphilis where the old and new descriptions are not coextensive.

The problem of semantic incommensurability has attracted different responses. One such response suggests that the descriptive theory of reference can still be preserved by narrowing down the theory dependence of terms. Bird (2004) and Dragulinescu (2011) make the distinction between thick and thin intensionalism. According to thick intensionalism, the theory dependence of a term's intension is very broad, such that many theoretical assumptions are included in it. According to thin intensionalism, the term's intension is much narrower, such that only some theoretical assumptions are included in it. They then suggest that the latter is not significantly affected by incommensurability, because narrow descriptions confer enough stability of meaning across changes in theory. However, while this may account for comparisons of terms across changes in the underlying theories, it is less clear whether it can account for such cases as Radden's cross-cultural comparison of Chinese and Western depression, where there are significant differences between descriptions that are exclusively symptom-based.

Another response to semantic incommensurability, and perhaps the most influential, appeals to Putnam (1975) and Kripke's (1980) causal theory of reference. This altogether denies that reference is determined by a description, instead proposing that it is determined by the nature of the phenomenon being investigated and its causal relation with the speaker. Kripke describes the processes of reference fixing and borrowing. Reference fixing involves the initial ostensive dubbing of a paradigmatic sample of the phenomenon by a speaker or group of speakers, such as the disease associated with neurodegeneration and progressive dementia being dubbed “Creutzfeldt-Jakob disease” in the 1920s. Reference borrowing involves the transmission of the dubbed term between speakers in the linguistic community via communicative exchanges.

Because reference determination depends on the nature of the phenomenon being investigated and not on the speaker's description associated with a term, the causal theory of reference offers a promising way around the problem of incommensurability. The description associated with “syphilis” has changed over the years, but the term's reference has not changed, because it is fixed by the initial ostensive dubbing of the sample. Changes in disease understanding do not result in changes in the term's meaning, but in better knowledge of the same disease and of what correctly belongs in the extensions of the term.

### 3.2 Disease kind essentialism

According to the causal theory, a term's extension is not determined by a description, but by the nature of the phenomenon in the external world. It is often argued that this implies a sort of essentialism, whereby a member of a kind has an essence that is necessary for its identity as a member of the kind (Haukioja, 2015, Putnam, 1975). A distinction is often made between intrinsic and relational essentialism. Intrinsic essentialism states that kind membership is determined by an intrinsic property of the phenomenon, such as its microstructure. For instance, the essence of water is its microstructure H_2_O, such that something must be H_2_O for it to be water. By contrast, relational essentialism states that kind membership is determined by a certain relation between the phenomenon and other phenomena, such as its causal history. For example, some philosophers argue that an organism's membership of a biological species depends on its phylogenetic lineage (LaPorte, 2004, Millikan, 1999).

Putnam (1975) assumes essentialism about disease kinds and, in doing so, supports a robustly causal conception of diagnostic terms. His position is expounded in detail by Williams (2011a). According to Williams, Putnam proposes that a disease has a relational essence, namely its cause. For example, the essence of polio is poliovirus infection, such that all and only instances of illnesses that involve poliovirus infection are cases of polio. A case of an illness that resembles polio in its symptom profile but which is not caused by poliovirus infection would not be a case of polio (Putnam, 1975, p.329). Conversely, an instance of poliovirus infection with atypical symptoms would still be a case of polio.

Note that one does not require prior knowledge of the nature of the cause of a disease to support essentialism. The cause is discovered *a posteriori*, but this does not change the reference of the diagnostic term, which is fixed by the dubbing of the paradigmatic sample. Even before the discovery of poliovirus, one could still consider the essence of polio to be its hidden causal structure and postulate that all cases of polio share the same kind of causal structure. The subsequent discovery of poliovirus elucidates the nature of this causal structure, and allows speakers to establish which cases have correctly and incorrectly been identified as cases of polio.

Williams also offers an analysis of Putnam's view on the causal relations between diseases and symptoms. First, Williams notes that Putnam rejects the descriptivist claim that a disease refers to a cluster of symptoms. For instance, Putnam states that “multiple sclerosis” does not mean “the simultaneous presence of such and such symptoms”, but “that disease which is normally responsible for some or all of the following symptoms…” (Putnam, 1975, p.329). Second, as noted above, Putnam claims that the essence of a disease is its cause, such as poliovirus being the essence of polio. Given that it is generally accepted in philosophy that causes are distinct from their effects, this suggests that although poliovirus is essential for something to count as a case of polio, it is nonetheless distinct from the disease state of polio itself.

This disease kind essentialism, then, assumes a causal chain with three components: “the cause of the disease, the disease itself, and the symptoms of the disease (which are caused by the disease)” (Williams, 2011a, p.167). For example, poliovirus causes polio, which in turn causes infantile paralysis. According to Williams' reading of Putnam, the cause of the disease is a relational essence that is distinct from the disease itself. This suggests that a disease term refers neither to the cause of the disease nor to the symptoms of the disease, but to an intermediate link in the causal chain: “polio” refers to the disease which is caused by poliovirus and which causes the symptoms of infantile paralysis.

The distinctions between the three steps in this causal chain can be interpreted as parallelling the distinctions between aetiology, pathology, and clinical features that are assumed in clinical textbooks: clinical features are the symptoms and signs with which the patient typically presents; pathology refers to the internal disease process that causes the clinical features; and aetiology refers to the more remote causal factors which are responsible for the pathology. For example, the entry on polio in Rendle-Short and Gray (1967, pp.386–387) states that the aetiology is the infectious organism poliovirus, the pathology is central nervous system destruction and muscle atrophy, and the clinical features include fever, malaise, and paralysis.

And so, an attraction of the three-step model is that it accommodates different kinds of causal explanatory talk in medicine. As noted above, the cause of the disease, the disease itself, and the symptoms of the disease are considered distinct nodes in a causal chain. This allows diseases to enter into causal explanations of symptoms, such as a case of infantile paralysis being explained by the diagnosis of polio and a case of paresis being explained by the diagnosis of syphilis. Moreover, it accounts for the way in which more general explanations of the diseases themselves appeal to their aetiologies, such as the disease polio being explained by poliovirus.

### 3.3 Some modifications

Although it has its merits, the analysis of disease terms presented here seems overly simplistic. As observed by Williams (2011a), the three-step model complements the germ theory of disease, according to which diseases are caused by pathogens and are classified on the basis of pathogen species. While this remains relevant for such infectious diseases as polio and syphilis, it is unsuitable for diseases that do not have specific singular causes but result from multiple contributing factors. Hence, the three-step model as it stands does not offer the most charitable rendering of the causal theory as applied to diagnostic terms. I now consider two modifications that help to address this.

The first modification involves relaxing the restrictions on the kinds of property that can constitute a disease essence. Putnam suggests that diseases have relational essences, namely their aetiological agents. However, as noted above many diseases do not have specific singular causes, and so are not classified on the basis of aetiological agent, but instead on the basis of pathophysiology. In some cases, this can be resolved by replacing relational essentialism with a kind of intrinsic essentialism, such that the essence of the disease is its pathophysiology. For example, the essence of appendicitis is inflammation of the appendix and the essence of bronchial carcinoma is uncontrolled cell growth in lung tissue.

However, this may not be quite enough in other cases where the pathophysiologies are more complex. Williams (2011b) cites rheumatoid arthritis (RA) as an example. He refers to the American Rheumatism Association's 1988 diagnostic criteria for RA, according to which the diagnosis is made if a patient displays at least four of seven anatomical, pathological, and radiological features. Each individual criterion is neither sufficient nor necessary for a diagnosis of RA. Moreover, different cases of RA may fulfil different combinations of criteria.

Williams concludes that RA does not have a simple essence. Rather, using a notion coined by Boyd (1999), he proposes that RA is best conceived as a homeostatic property cluster (HPC). According to this view, members of a given kind do not have to share a single necessary property, but can share clusters of similarities that are causally connected. For example, Boyd suggests that biological species are HPCs. Members of a species share a number of common properties, but there is significant variation within the species that no single property is essential for membership within that species. Similarly, Williams proposes that there is a cluster of properties that can be satisfied to varying degrees for something to be a case of RA, but it is neither sufficient nor necessary for any particular one of these properties to be satisfied. This potentially allows for more variability between the members of a kind, as different combinations of the properties may be satisfied for kind membership.^2^

The second modification involves expanding the three-step causal chain into a more complex causal network. As noted by Thagard (1999), disease causation is usually a complex process with multiple interplaying factors. Not only can there be numerous risk and protective factors that influence the development of the disease, but the disease itself can be a causal factor that influences the development of other diseases. This suggests that disease causation cannot be adequately modelled by a simple linear chain. Rather, a more complex causal network is needed to acknowledge the multifactorial aetiologies of some diseases.

**Fig. 1:**
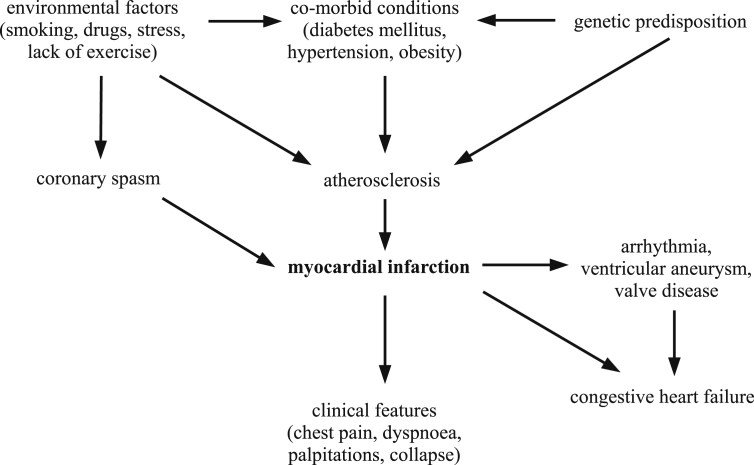
Causal network for myocardial infarction.

Fig. 1 shows an example of a causal network for myocardial infarction. This network acknowledges multiple aetiological factors, including other diseases that contribute to the development of myocardial infarction. It also includes other diseases caused by myocardial infarction.

Due to the acknowledgement of multiple factors involved in disease causation, the causal network model is overall more satisfying than the three-step model. Again, this is fully compatible with the causal theory of reference and a causal conception of diagnostic terms: “myocardial infarction” refers to the pathology that causes a patient's clinical features. However, rather than just being the intermediate link in a linear causal chain, the model acknowledges that myocardial infarction is nested within a broader causal network and has complex causal connections with other diseases.

According to Thagard (1999, p.114), the causal relations in such a network are intended to map onto the actual causal relations in individual cases of the disease. However, not every feature of the network has to be present in every instance of the disease. Thagard states that the causal relations in the model are not deterministic, but statistically-based. Hence, different instances of myocardial infarction may result from different combinations of aetiological factors. This seems to support HPC theory, but is also consistent with the sort of intrinsic essentialism where the essence of the disease is its pathophysiology. For instance, it could be claimed that the essence of myocardial infarction is necrosis of the myocardium from prolonged ischaemia, but different cases could differ with respect to what had caused this ischaemic necrosis.

### 3.4 Merits of the causal theory

To summarise this section, I presented the causal theory of reference as an account of how the reference of a diagnostic term is determined. According to this theory, it is not determined by a description, but by the actual nature of the disease and the causal relations between speakers who use the term. For Putnam, it is not the symptoms of a disease, but its cause that is essential for the individuation of meaning. The resulting essentialism implies a disease model consisting of three parts: the cause of the disease; the disease itself; and the symptoms. However, I argued that this model is too simplistic and suggested two modifications that allow a more charitable rendering of the causal theory. One modification, after Williams, is to allow HPCs as well as simple essences as determinants of reference. The other modification, after Thagard, is the expansion of the three-step chain into a more complex causal network.

The causal theory of reference supports a robustly causal conception of diagnostic terms, according to which diagnostic terms do not refer to sets of symptoms, but to the disease processes that cause the symptoms. This is the case even before the precise natures of these disease processes are fully known, as these can be discovered *a posteriori*. As noted in subsection 3.1, this helps to avoid the problem of incommensurability that affects the descriptive theory. Because reference is not determined by a description, the change in description that results from changing disease understanding do not amount to a change in reference: “syphilis” did not go from referring to a set of characteristic symptoms to referring to *T. pallidum* infection, but has referred to the same disease from the outset. The subsequent discovery of *T. pallidum* simply increased our knowledge of the nature of this disease.

In addition, these causal considerations explain why certain collections of symptoms are characterised by doctors and scientists into distinct syndromes. As noted by Williams (2011b), such actions are those of people who consider the associated symptoms to be connected by a unifying causal structure. In such case as syphilis, it turns out that the symptom cluster is actually the result of a singular kind of pathology. In other cases, it turns out that there are multiple different pathologies, each of which can cause the observed cluster of symptoms. For example, “dropsy” had been used for many centuries to refer to the alleged disease associated with fluid retention. However, it turned out that there are multiple different pathologies that could underlie cases of dropsy, and so the term was discarded and replaced by more specific diagnostic terms, such as “nephrotic syndrome”, “congestive heart failure”, and “cirrhosis of the liver” (Peitzman, 2007).

According to the account presented in this section, a psychiatric diagnosis, such as “major depressive disorder”, does not refer to a cluster of symptoms, but to the disease process that causes these symptoms. This accounts for the way in which clinical texts use diagnostic terms in psychiatry to refer to the causes of symptoms, as noted in Section 1. Furthermore, it provides a possible solution to problem of cross-cultural incommensurability presented by Radden's comparison of Chinese and Western depression in subsection 2.3. If “depression” is taken to refer to the putative disease process that produces various symptoms, then Chinese and Western depression can be considered to be the same disorder based on the assumption that they both involve this same disease process, despite their having different symptom profiles.

However, in spite of these attractions, I argue that a pure causal theory has significant shortcomings regarding psychiatric diagnoses. In particular, I argue that by supporting robustly causal conceptions of psychiatric disorders, it downplays the important functions of their symptom-based diagnostic criteria in such manuals as DSM-5. Section 4 examines this in more detail and proposes a two-dimensional semantic framework that preserves the core features of the causal theory while taking the descriptive diagnostic criteria seriously.

## 4 Two-dimensional semantics

### 4.1 Diagnostic criteria in psychiatry

As noted in Section 3, a key premise of Putnam and Kripke's causal theory of reference is that the reference of a term is not determined by a description of superficial properties. Accordingly, Putnam argues that descriptions of symptoms are not necessary to the meanings of diagnostic terms. Rather, they constitute stereotypes, which provide conventional ideas of what the disorders look like but are not analytically tied to their associated terms. This does seem to be plausible for some of the medical diagnoses mentioned throughout this paper, where the symptoms appear to be contingent properties of the diseases. A painless case of inflammation of the appendix is still a case of appendicitis. However, diagnostic terms in psychiatry, such as “panic disorder” and “delusional disorder”, do seem to allude to their symptoms in ways that suggest more fundamental connections between the symptoms and the disorders. It seems oxymoronic to claim that a person could have panic disorder without having recurrent panic attacks, or that a person could have delusional disorder without having delusions.

In response, the causal theorist could appeal to the difference between connotation and denotation. Kripke (1980, p.26) uses the example of the town, Dartmouth. The name may have the connotation of a location at the mouth of the River Dart, but this is not its denotation. According to Kripke, the town could still retain the name “Dartmouth”, even if the River Dart changes its course, and so the connection between Dartmouth and its location at the mouth of the River Dart is contingent. Similarly, one might claim that “panic disorder” has the connotation of certain symptoms, but denotes the underlying disease that usually causes these symptoms.

However, this analogy is not wholly accurate. What it does not acknowledge is that the symptom-based definitions in DSM-5 are not just descriptions of disorders, but necessary criteria for applying the diagnostic terms. While Dartmouth may still retain its name if the River Dart changes course, the DSM-5 criteria for panic disorder preclude a diagnosis of panic disorder unless recurrent panic attacks are present. They set the conditions that something must satisfy for it to qualify as an instance of the diagnosis. Hence, an analysis of the reference of such a diagnostic term as “panic disorder” would need to account for the fact that the presence of the relevant symptom cluster is necessary for the correct application of the diagnostic term. Again, this is unlike the case of a medical diagnosis such as acute appendicitis, where the presence of the stereotypical symptoms is not necessary for the diagnosis to be applied.

It appears that a pure causal theory is not adequate for the analysis of diagnostic terms in psychiatry, because it relegates the symptom-based diagnostic criteria to mere contingent features of the disorders. This contradicts the important functions of these symptom criteria as necessary conditions for applications of the diagnostic terms. However, for reasons highlighted in Section 2, a return to pure descriptivism would be undesirable. In what is to follow, I show how the framework of two-dimensional semantics can help.

### 4.2 An overview of the framework

Before I consider its application to diagnostic terms specifically, I want to lay out the general motivations for two-dimensional semantics in more detail. As previously noted, the causal theory of reference states that the reference of a term such as “water”^3^ is not determined by a description of water's superficial properties, but by what turns out to be its essence, namely the microstructure H_2_O. According to the causal theory, then, “water” has a single intension, which rigidly designates H_2_O. This is taken to apply across all possible worlds, such that “water = H_2_O” is necessarily true. However, as noted by Chalmers (1996), there remains an intuition that “water” and “H_2_O” differ in some aspect of meaning. The two are not epistemically equivalent. For instance, one could know that the potable liquid found in rivers is water, and not know that it is H_2_O. Furthermore, although the potable liquid found in rivers which speakers had dubbed “water” actually did turn out to be H_2_O in our world, we can still entertain a hypothetical scenario in which this liquid we call “water” was discovered to be something else.

These intuitions suggest that there is more to the meaning of “water” than having the microstructure H_2_O. In the literature (Chalmers, 1996, Jackson, 1998), this is normally explicated in modal terms with a retelling of Putnam's (1973) Twin Earth thought experiment. Twin Earth is indistinguishable from Earth in almost every way. Like Earth, its rivers contain a colourless, tasteless, and potable liquid, which its inhabitants call “water”. The difference between the two worlds is that the stuff we call “water” on Earth was discovered to have the microstructure H_2_O, whereas the corresponding stuff on Twin Earth that is called “water” by its inhabitants was discovered to have the microstructure XYZ. According to the causal theory of Kripke and Putnam, this Twin Earth liquid is not water. Because “water” designates rigidly and has been shown by chemistry to pick out H_2_O on Earth, “water = H_2_O” is a necessary truth that holds across all worlds. Hence, Twin Earthlings are wrong to claim that “water = XYZ”. However, if a causal theorist from Twin Earth were to apply the same standards, then he or she would arrive at the opposite conclusion and claim that we Earthlings are wrong to call our liquid “water”, because the liquid that Twin Earthlings had dubbed “water” was shown by chemistry to be XYZ. This suggests that if the world had turned out to be like Twin Earth, then “water” would refer to XYZ instead of H_2_O.

The above brings out the tension between the causal theorist's claim that “water” necessarily refers to H_2_O and the intuition that it could have referred to something else had the world turned out to be different in the relevant way. Two-dimensional semantics resolves this tension. This is a formal framework developed by Stalnaker (1978), and later championed by Jackson (1998) and Chalmers, 1996, Chalmers, 2010.^4^ It proposes that the meaning of “water” is not only dependent on *a posteriori* facts about the world, but also on which possible world we assumed to be the actual world in which the reference is fixed. In the framework of Kripke and Putnam, only Earth is taken to be actual, while all other possible worlds are taken to be counterfactual. Hence, in this scenario, “water” only picks out the substance that was discovered to be H_2_O. However, if one assumes Twin Earth to be the actual world that one inhabits, then “water” would pick out the substance that was discovered to be XYZ.

Two-dimensional semantics, then, proposes that a given term, such as “water”, is taken to express two intensions.^5^ The primary intension (1-intension) of a term is what the term would pick out in a chosen world if that world is imagined to be the actual world in which the reference is fixed. Given that the reference fixing occurs before the discovery of the underlying essential nature of the phenomenon in question, the 1-intension roughly approximates to the phenomenon's pre-theoretical mode of presentation.^6^ For instance, the 1-intension of “water” (1-WATER) roughly corresponds to the colourless, tasteless, and potable liquid found in rivers. As mentioned above, this liquid that was dubbed “water” turns out to be H_2_O in the scenario where Earth is imagined to be the actual world in which the reference is fixed, but turns out to be XYZ in the scenario where Twin Earth is imagined to be actual.

The secondary intension (2-intension) of a term is what the term picks out if we fix our world as actual and then evaluate other worlds as counterfactual relative to it. The 2-intension of “water” (2-WATER) only picks out H_2_O, because water was discovered to be H_2_O in our world. This identity is taken to be necessary, such that “water” refers to H_2_O across all counterfactual worlds. Note that the 2-intension corresponds to the Kripkean intension as per the causal theory of reference. The reference of 2-WATER is not determined by a cluster of descriptions, but by the microstructure H_2_O. Therefore, two-dimensional semantics assimilates the causal theory of reference. Along with it, I argue that it can assimilate the modifications to the causal theory presented in subsection 3.3, such that HPCs as well as simple essences may be the determinants of 2-intensions.

The above modal story accounts for the way in which there can be two dimensions of a term's meaning, one which is dependent on the pre-theoretical mode of presentation of the phenomenon associated with the term, and another which is dependent on what the underlying nature of this phenomenon *a posteriori* turns to be. It also has implications for the notions of necessity and contingency. These implications are useful for understanding the conceptual relations between a term and its associated concepts. For example, the sorts of relation that “water” has with “H_2_O” and with “potable liquid found in rivers” depend on whether we assume 1-WATER or 2-WATER. If 1-WATER is assumed, then “water = potable liquid found in rivers” is necessarily true, because it is defined by this mode of presentation, while “water = H_2_O” is contingently true, because the potable liquid that was dubbed “water” in another world could have turned out to be something other than H_2_O. On the other hand, if 2-WATER is assumed, then “water = H_2_O” is necessarily true, because the liquid that was dubbed “water” in our world *a posteriori* turned out to be H_2_O, while “water = potable liquid found in rivers” is contingently true, because H_2_O could have had a different form in a counterfactual world.

This can be captured by the idea that there are two sorts of necessity and two sorts of contingency, which respectively correspond to the necessity and contingency when the 1-intension of a term is assumed (1-necessity and 1-contingency), and to the necessity and contingency when the 2-intension of the term is assumed (2-necessity and 2-contingency) (Chalmers, 2010, p.167). Hence, “water = potable liquid found in rivers”, is 1-necessary but 2-contingent, whereas “water = H_2_O” is 2-necessary but 1-contingent. 1-necessity can be thought of as corresponding to the definitional relation between a term and a description, while 2-necessity corresponds to the Kripkean *a posteriori* necessity as per the causal theory of reference. As I shall show in the following subsection, this offers a way of analysing the conceptual relations between psychiatric diagnoses, DSM-5 symptom criteria, and the pathologies purported to cause these symptoms.

And so, the two-dimensional semantic framework presented here can be thought of as one way of synthesising the causal theory with descriptivist considerations to capture different aspects of a term's complex semantic value that have useful epistemic roles (Chalmers, 2010, p.563). In virtue of a term's 2-intension, a causal theorist can accept Chalmers' account of how the reference of that term is determined. However, while the causal theorist might consider this to constitute the entire meaning of the term, for Chalmers it only constitutes one aspect of its meaning. The term also has a 1-intension that corresponds to its mode of presentation. An implication of this is that the two-dimensional theorist can reject the causal theorist's claim that a definition based on superficial properties is not relevant to the reference of a term. A definition is not merely a stereotype, but is a genuine aspect of a term's meaning. As we shall see, this makes two-dimensional semantics better suited than a pure causal theory for the analysis of diagnostic terms in psychiatry.

### 4.3 A two-dimensional semantic account of diagnostic terms

I propose that a two-dimensional semantic framework can make sense of terms whose applications necessitate the presence of certain superficial properties despite the terms being used by speakers to refer to the causes of these properties. This is particularly relevant to psychiatry, where the meanings of diagnostic terms are necessarily tied to descriptions of symptoms despite the terms being invoked to refer to the causes of these symptoms. Although this particular issue is not so obviously a problem for many diagnoses in somatic medicine, the framework can nonetheless accommodate the analysis of medical diagnoses as well.

In subsection 4.2, I explicated the principles of the framework with appeal to the classic example of “water”. I suggest that diagnostic terms are amenable to the same kind of analysis. Like “water”, a diagnosis has a pre-theoretical mode of presentation that characterises the 1-intension, and an underlying structure that is discovered *a posteriori* and determines the 2-intension. In the case of “water”, the mode of presentation is roughly the colourless, tasteless, and potable liquid found in lakes and rivers, whereas the *a posteriori* discovered underlying structure is H_2_O. In the case of a diagnosis, the mode of presentation is the clinical manifestation and the underlying structure is the disease process that is responsible for the clinical manifestation. For example, the 1-intension of the term “polio” is roughly the transmissible condition presenting with infantile paralysis, whereas the 2-intension is poliovirus infection.

In psychiatry, the clinical manifestations for diagnoses are codified in the DSM-5 definitions. A DSM-5 definition, then, captures the 1-intension of a diagnosis. For example, the 1-intension of “panic disorder” includes “recurrent unexpected panic attacks” (APA, 2013, p.209) and the 1-intension of “delusional disorder” includes “the presence of one or more delusions that persist for at least 1 month” (APA, 2013, p.92). As previously mentioned, these definitions serve as necessary criteria for the diagnoses, which squares neatly with Chalmers' (2002, p.143) suggestion that the role of a description is to provide conditions that give speakers ways to identify the extension of the term. The 2-intensions of diagnoses are whatever turn out to be their respective underlying pathological processes, as per the causal theory of reference.

It is worth noting here that the 1-intensions may change across time, as demonstrated by the changes in the criteria for schizophrenia from DSM-IV to DSM-5 mentioned in Section 2.2. This is compatible with Chalmers' account, as he accepts that certain kinds of conceptual change involve changes in an expression's 1-intension (Chalmers, 2012, p.210). However, the implication of semantic incommensurability is avoided here by the supposition that the 2-intension is invariant. Hence, although DSM-IV schizophrenia and DSM-5 schizophrenia have different 1-intensions, they are assumed to have the same 2-intension in virtue of their referring to the same causative pathology. This highlights the point made in Section 2.2 that diagnostic terms also go through sorts of conceptual change other than the changes from descriptive to causal conceptions suggested by Hempel and Thagard. In particular, they can undergo changes in the descriptive definitions, which amount to changes in their 1-intensions.

Furthermore, Chalmers also suggests that it is possible at a given time for concepts to have different 1-intensions but the same 2-intension. For example, “Hesperus” and “Phosphorus” have different 1-intensions, as the former picks out the evening star and the latter picks out the morning star, but they have the same 2-intension, as both refer to the planet Venus (Chalmers, 1996, p.65). I suggest that this can be applied to Radden's cross-cultural example of Chinese and Western depression. The differences in the symptom profiles of Chinese and Western depression can be taken to constitute different 1-intensions, but the two can still be equated on the basis of the assumption that they have the same 2-intension, hence avoiding the implication of cross-cultural semantic incommensurability.

Analysing diagnostic terms in psychiatry as having 1-intensions and 2-intensions allows us to take their descriptive definitions in DSM-5 seriously as necessary criteria making the diagnoses, yet still talk about the diagnoses as referring to the causes of the symptoms that make up these definitions. Let us consider, for example, the connection between “panic disorder” and the DSM-5 description “recurrent unexpected panic attacks”. If the 1-intension of “panic disorder” is assumed, then the connection is necessary, because the 1-intension is defined through this DSM-5 description. This 1-necessity reflects the way in which the DSM-5 symptom criteria are explicitly required for the diagnosis to be made. A diagnosis of panic disorder, for instance, cannot be made unless recurrent unexpected panic attacks are present. Therefore, unlike a pure causal theory of reference, two-dimensional semantics does not relegate the DSM-5 symptom criteria to contingent features of the disorder, but acknowledges they are necessarily tied to the diagnosis in virtue of the diagnostic term's 1-intension.

If the 2-intension of “panic disorder” is assumed, then the term refers to whatever turns out to be the pathology that normally causes recurrent unexpected panic attacks. The connection between the 2-intension of “panic disorder” and “recurrent unexpected panic attacks” is contingent, because it is counterfactually conceivable that the pathology picked out by the 2-intension could be present without it being accompanied by recurrent unexpected panic attacks, just as it is conceivable for inflammation of the appendix to be present without it being accompanied by abdominal pain. This reflects the way in which the diagnostic term is used in medical textbooks and health information resources to refer to what is causing a set of symptoms, such as panic disorder being invoked as the cause of a patient's panic attacks.

A two-dimensional semantic analysis, then, provides a resolution to the tension between the DSM-5 definitions of psychiatric diagnoses as symptom clusters and their uses in other clinical texts as terms that refer to the causes of these symptom clusters. Under this framework, a diagnostic term does not have a single intension, but a complex semantic value involving a 1-intension and a 2-intension. These two intensions have different epistemic roles that capture the two kinds of talk mentioned above. In virtue of their 1-intensions, diagnostic term are defined through their symptoms, which captures their symptom-based definitions in DSM-5. In virtue of their 2-intensions, they refer to the pathologies that normally cause these symptoms, which captures their uses as explanations of patients' symptoms in textbooks and health information resources. This suggests, *pace* Szasz, that although a psychiatric diagnosis is defined through its symptoms, this does not necessarily preclude it from serving as a causal explanation of these symptoms.

### 4.4 Other implications

As well as resolving the tension between the two kinds of talk regarding psychiatric diagnoses, two-dimensional semantics has further strengths as a framework for the analysis of diagnostic terms more generally. First, its semantic pluralism helps to characterise the different kinds of information conveyed by diagnostic terms in the communicative exchanges of clinicians. A diagnosis normally provides both information about a patient's likely clinical presentation and information about the underlying disease process, which respectively correspond to the 1-intension and 2-intension of the diagnostic term. For example, “chronic bronchitis” not only informs the clinician that the patient is likely to be presenting with cough and sputum expectoration, but also that the underlying disease process is inflammation of the airways.

Second, two-dimensional semantics not only provides a way of interpreting changes in disease understanding that does not imply radical incommensurability, but also has the added advantage of taking seriously the different epistemic possibilities entertained by scientists in the early stages of disease understanding. Before the nature of the underlying pathology is understood, speakers rely on the 1-intension of the disease term. For example, before poliovirus was discovered by Landsteiner and Popper in 1909, doctors applied the term “polio” to cases of infantile paralysis, while aiming to elucidate the underlying causal structure. This 1-intension analysis allows for the intuition that the condition presenting with infantile paralysis that was dubbed “polio” could have turned out to be caused by something else had the world been different in the relevant way. In actuality, polio turned out to be caused by poliovirus, which indicates that “polio = poliovirus infection” is 2-necessary. However, before poliovirus was discovered, Landsteiner and Popper had initially tried to look for a responsible bacterial agent (Skern, 2010, p.1372). This suggests that they had entertained the epistemic possibility that polio is not caused by poliovirus infection, which can be captured with the analysis that “polio = poliovirus infection” is 1-contingent.

Once the nature of polio's underlying pathology was discovered to be poliovirus infection, speakers could then utilise the 2-intension, which is determined by rigidifying this evaluation so that “polio” refers only to poliovirus infection across all worlds. Because the 2-intension fixes the reference of “polio” across all worlds, it establishes which cases of infantile paralysis are cases of polio and which ones are not. Hence, cases of infantile paralysis in the past that were not caused by poliovirus infection were not actually cases of polio. It is this 2-intension that is central to the aims of further scientific research into prevention and treatment. When Salk and Sabin were developing vaccines for polio, they were developing vaccines specifically to prevent poliovirus infection.

The above suggests that the move from descriptive to causal conceptions of a diagnostic term involves the change in emphasis from the term's 1-intension to its 2-intension. This does not involve the semantic incommensurability permitted by the descriptive theory of reference, because the determination of a diagnostic term's reference follows the same processes of reference fixing and borrowing as proposed by the causal theory of reference. The 2-intension of the term rigidly designates what the causal structure of a disease turns out to be, which maintains reference stability. Hence, “polio” denotes only genuine cases of poliovirus infection. Nonetheless, the 1-intension accounts for the epistemic possibility of scenarios where the condition that was dubbed “polio” had turned out to be caused by something other than poliovirus.

## 5 Summary and further considerations

This paper has explored how philosophical theories of reference apply to diagnostic terms, with the aim of resolving the tension between the descriptive definitions of psychiatric diagnoses in DSM-5 and their causal conceptions in other clinical resources. I argued that there are good reasons to reject a traditional descriptive theory of reference regarding diagnostic terms, including the implication of semantic incommensurability between old and new uses of a term. Although the causal theory of reference fares better at maintaining reference stability and complements causal conceptions of diagnostic terms, I argued that a pure causal theory does not account for diagnoses whose criteria specify the fulfilment of particular symptom descriptions. I sketched how a two-dimensional semantic framework that assimilates the causal theory with descriptive considerations resolves this problem and accommodates the two seemingly contradictory ways in which diagnostic terms are used in psychiatry.

The framework I have presented suggests that invoking psychiatric diagnoses as causal explanations of patients' symptoms is not necessarily precluded by the fact that they are defined through these symptoms. This partly addresses the Szaszian worry that a mental illness cannot explain behaviour because it is just a shorthand label for this behaviour. However, some concessions need to be made, which I consider in this final section.

First, for some disorders, there are doubts about whether the underlying causal structures will turn out to be sufficiently stable and repeatable for their respective diagnostic categories to be considered epistemically useful. In other words, it may turn out to be the case that the symptoms of the disorder can be produced in many different ways and that there is no unifying set of mechanisms that is shared by every instance of the disorder. Research into the pathophysiology of major depressive disorder (MDD) has not revealed any single underlying cause, but many diverse factors at multiple levels, none of which are universally present across all cases (Hasler, 2010). The worry, then, is that “MDD” could turn out to be like the case of “dropsy” mentioned in subsection 3.4, such that its 2-intension refers to a disjunct of cause_1_ or cause_2_ or cause_3_ or … or cause_n_ of such and such symptoms, or any combination thereof. Perhaps “MDD” could be discarded in favour of more precise terms, each of which refers to each of the causal structures that usually produce the typical symptoms. However, it may be the case that the vast number of different interacting causal factors implicated would make such a neat subdivision problematic.

Second, the framework I present does not offer an account of the social processes that also influence the semantic practices surrounding psychiatric diagnoses. One such account of these processes in the philosophical literature is Hacking's (1999) theory of dynamic nominalism. Using the example of childhood autism, Hacking (1999, pp.114–115) proposes that psychiatric disorders are interactive kinds: categorising disorders results in looping effects that alter the natures of the disorders in question. He argues that since the category of childhood autism was coined, the ideas about the disorder that became prevalent in society have influenced the sorts of behaviour with which new cases present. This suggests that there is an aspect of “childhood autism” that changes in response to social processes.

I propose that this complements rather than challenges the two-dimensional semantic framework I have presented. In fact, Hacking (1999, pp.119–124) himself is sympathetic towards the use of Putnam and Kripke's causal theory as a tool to analyse the semantics of diagnostic terms, such as “childhood autism” being used to designate the putative pathology *P*. This suggests that childhood autism is an interactive kind with respect to its prototypical symptoms, but is presumed to be a natural kind with respect to *P*. I argue that this is consistent with the analysis that the changes that result from looping effects are with respect to the 1-intension of “childhood autism”, whereas the 2-intension is posited as remaining stable in virtue of *P*. Of course, it may turn out that *P* is a range of pathologies rather than a single definite pathology, but this too can be accommodated with the analysis that the 2-intension of “childhood autism” is disjunctive. Nevertheless, Hacking's important observations highlight that there are dynamics working at the level of classification that are not specifically expounded by the theories of reference discussed in this paper. And so, while the two-dimensional semantic framework presented in this paper accounts for the semantic practices of psychiatrists working within a biological programme of research, a more complete analysis of the meanings of psychiatric diagnoses would also need to consider the historical and social processes involved in meaning-making.

## Footnotes

1 Thagard (1999, pp.118–134) describes four stages of disease understanding: (1) disease characterisation, which involves the clustering together of a set of associated symptoms; (2) cause specification, which involves the observation of factors correlated with the disease and postulating them as possible aetiologies; (3) experimentation, which involves the gathering of evidence to support a causal hypothesis; (4) elaboration of mechanisms linking the aetiology of the disease to its manifestations.

2 Although Williams' suggestion that some diagnostic terms are determined by HPCs rather than simple essences is plausible, I argue that such a move may not be necessary in his given example of RA. While not completely understood, the pathophysiology of RA is tidier than Williams acknowledges, being characterised by the autoimmune reaction to autoantigens expressed in the joints (Boissier, Semerano, Challal, Saidenberg-Kermanac'h, & Falgarone, 2012). Therefore, the essentialist could justifiably claim that the essence of RA is the autoimmune damage to the joints.

3 I am grateful to anonymous referee who raised a concern about the suitability of the “water” example, considering that the framework presented here is proposed as a solution to a problem regarding diagnostic terms. In response, my aim in this subsection is to explicate the framework by presenting a familiar paradigm case where it has been applied and from which parallels can be drawn to the cases of diagnostic terms. Not only is “water” the example which has received perhaps the most detailed analysis by proponents of two-dimensional semantics (Chalmers, 1996, Jackson, 1998), but it is an example that has been used to demonstrate a problem with the causal theory of reference similar to the problem I suggest is presented by certain diagnostic terms. As I note in subsection 4.1, it is counterintuitive to think of a case of panic disorder without panic attacks. Similarly, it has been argued that notwithstanding the causal theory of reference, the superficial properties of water are still somehow relevant to the meaning of “water” (Barnett, 2000).

4 Different proponents of two-dimensional semantics apply it to different problems. Stalnaker (1978) uses it to examine what is communicated by speakers when there is doubt about the meanings of certain utterances. Jackson (1998) uses it to reconcile semantic externalism with an *a priori* approach to meaning. Chalmers, 1996, Chalmers, 2010 famously uses it to endorse dualism in the philosophy of mind.

5 Different authors use different names for these two intensions, but here I follow the terminology of Chalmers (2010).

6 Chalmers (1996, pp.56–65) initially suggests that the 1-intension may be determined by a Fregean description of its referent. However, he later concedes that this is not the case for all 1-intensions, as it is doubtful whether some terms, such as “knowledge”, can be adequately encapsulated in a description (Chalmers, 2002, pp.143–149). Instead, he suggests that 1-intensions can be thought of as capturing what the extensions of terms would be in different epistemic possibilities about how the world turns out. While these may sometimes be captured by descriptions, this need not always be the case.
